# Using motivational interviewing to reduce parental risk related behaviors for early childhood caries: a pilot study

**DOI:** 10.1186/s12903-020-1052-6

**Published:** 2020-03-29

**Authors:** Christine M. Blue, Michelle C. Arnett, Hiwet Ephrem, Scott Lunos, Chen Ruoqiong, Robert Jones

**Affiliations:** 1 Division of Dental Hygiene, University of Minnesota, 515 Delaware Street SE, 9-372 Moos Tower, Minneapolis, MN 55455 USA; 2 Division of Dental Hygiene, University of Minnesota, 420 Delaware St SE, MMC 729, Minneapolis, MN 55455 USA; 3 University of Minnesota, 717 Delaware Street SE, 140-27, Minneapolis, MN 55414 USA; 4 Department of Diagnostic and Biological Sciences, University of Minnesota, 515 Delaware Street SE, 17-237 Moos Tower, Minneapolis, MN 55455 USA; 5 Division of Pediatric Dentistry, University of Minnesota, 515 Delaware Street SE, 6-150 Moos Tower, Minneapolis, MN 55455 USA

**Keywords:** Oral health, Motivational interviewing, American Indian children, Early childhood caries

## Abstract

**Background:**

Decades of epidemiological studies have documented high rates of early childhood caries (ECC) among American Indian and Alaska Native (AIAN) children. The aim of this pilot study was to investigate if a motivational interviewing (MI) intervention improved oral self-care behaviors of AIAN caregivers of infants, and determine if the MI intervention promoted positive changes in caregivers’ ECC risk-related behaviors.

**Methods:**

Caregivers of infants presenting for well- child visits in a medical clinic were randomized to treatment and control groups. At the first visit, a caries risk test (CRT) for cariogenic bacteria was completed for both groups. The Parental Care of Child’s Teeth (PCCT) was administered at the second visit and used to assess ECC risk-related behaviors. Over the course of four well-child visits, caregivers in the treatment group participated in a MI discussion focusing on behavior changes and desired outcomes for their personal oral health and their child’s. The duration of the intervention was 1 year. The control group was given oral health information traditionally provided at well-child visits. At the fourth well-child visit, the CRT and PCCT questionnaire were administered again.

**Results:**

The mean bacterial load for mutans streptococcus (MS) was similar at both visits. A slight reduction in the mean bacterial levels of lactobacilli was observed in both the test and control groups after the last visit, although not at a level of statistical significance. The treatment group showed minimal improvement in child feeding practices and nighttime bottle habits.

**Conclusions:**

Motivational Interviewing had little effect on oral self-care behaviors as measured by bacterial load, nor did MI reduce parental risk related behavior for early childhood caries.

**Trial registration:**

Clinicaltrials.gov# NCT04286256. Retrospectively registered, February 26, 2020.

## Background

ECC is defined as the presence of decayed, missing, or filled tooth surfaces of primary teeth in children up to 6 years of age or younger [1]. ECC is a transmissible infectious disease caused by pathogenic bacteria including, but not limited to mutans streptococci (MS) and lactobacilli (LB). Poor oral hygiene at home and/or lack of fluoride increases the risk for ECC [2–4]. Disease transmission is also rooted in the behavior of parents or primary caregivers who expose children to cariogenic microorganisms through intimate contact of saliva by sharing and/or tasting foods and/or pacifier contamination [2–4]. Decreasing the level of cariogenic organisms in the mother’s/primary caregiver’s oral flora at the time of colonization can significantly reduce a child’s predisposition to ECC [3]. Additionally, cariogenic organisms have been used as outcome measures to assess oral self-care behaviors and reduce ECC [5, 6]. Parental/caregiver behaviors play an instrumental role in defining oral health practices early in a child’s life, including establishing regular dental care [7]. Therefore, caregiver beliefs, attitudes, self-efficacy and social circumstances will impact the engagement of oral health-promoting behaviors that influence ECC development [3, 7].

Despite the recent increase of children enrolled in Medicaid, utilizing preventive oral health care is still a challenge [8]. As a response to the access to care issue, Minnesota passed legislation in 2009 allowing the education and licensing of dental therapists (DT). The intent of this legislation was to address oral health disparity by creating an oral health provider who could expand access to dental care in Minnesota. A DT may provide preventive, as well as most basic restorative procedures to children and adult patients [9]. The law stipulates that a DT must work in “practice settings that serve the low-income and underserved.” The University of Minnesota (UMN) School of Dentistry (SOD) enrolled its first DT class in September 2009 and has since graduated over 60 dental therapists.

The prevalence of untreated caries among American Indian and Alaska Native (AIAN) children was reported higher compared to any other racial or ethnic group in the US [10, 11]. AIAN parents face barriers (e.g., distance to treatment, lack of transportation, shortage of dental clinics offering culturally responsive care, and costs) that can limit their access to professionally applied preventive services (e.g., prophylaxis, fluoride varnish) and restorative care for their children [10, 11]. According to Schroth and colleagues, efforts to improve oral health in indigenous children need to be informative, non-judgmental, and culturally appropriate [11]. While some clinical trials involving indigenous children have used chemotherapeutic agents to prevent or arrest caries, other investigations using multi-pronged approaches that address social determinants of indigenous children’s health have shown promise [11, 12].

Essential to the success of oral hygiene programs are evidence-based behavior change models which have only recently been considered in dentistry [13–15]. MI is a patient-centered, collaborative counseling approach designed to strengthen an individual’s intrinsic motivation towards a positive behavior change [16]. Motivational interviewing (MI) has emerged as a successful strategy to address undesirable behaviors such as unhealthy eating habits, lack of exercise, and smoking and alcohol use [17–19]. MI focuses on an individual-provider partnership and is based on the premise that an individual’s reasons for change and autonomy to make their own decisions should be supported by the provider [16]. MI has shown different degrees of effectiveness in preventing ECC in clinical trials involving pregnant women and mothers of young children [14, 15]. Harrison et al., found preliminary evidence that MI-style interventions had an impact on the severity of caries in indigenous children in Quebec, Canada [14]. Behavioral interventions and effective communication between a parental/caregiver and an oral health provider may improve a parental/caregiver’s decision-making with regard to risk-related behaviors for ECC [14].

The study’s aim was to investigate if a MI intervention improved oral self-care behaviors of AIAN caregivers of infants, and determine if the MI intervention promoted positive changes in caregivers’ ECC risk-related behaviors. Objective 1 was to determine if individualized MI sessions with a student DT improved the oral self-care behaviors of test participants measured by reduction in oral bacterial load [3, 4, 6]. Given a reduction in the level of cariogenic organisms in a caregiver’s oral flora at the time of colonization can significantly reduce a child’s predisposition to ECC, the hypothesis for objective 1 was, there is no difference in oral bacterial load (MS and LB), as measured by the CR, between test participants and control participants. Objective 2 was to determine if the MI sessions by the student DT promoted positive changes in test participants’ ECC risk-related behaviors. The hypothesis for objective 2 was, ECC risk-related behaviors of test participants are no different than those of control participants.

## Methods

A randomized, controlled pilot study was approved by the UMN Institutional Review Board (IRB) #1107 M02642. Eligibility for participation in this study included healthy caregivers (> 18 years of age) of infants (< 1 year of age). Exclusion criteria included antibiotic use within the past 3 months, immunosuppressive medications, and participants who self-reported xerostomia due to the possible effect on the oral flora.

Data collection for this study occurred at well-child visits at the Native American Community Clinic (NACC), located in Minneapolis, Minnesota during 2012–2013. The NACC serves as one of the UMN School of Dentistry’s (SOD’s) outreach clinics and provides experiential education rotations for dental, dental hygiene, and DT students. Dental, medical, and mental health services are provided to an ethnically diverse community with many underserved health care needs. NACC is a non-profit Community Health Center/Federally Qualified Health Center (FQHC) that has been serving Native Americans and others in the Twin Cities metropolitan area since 2003. Currently, 85% of the patient population at NACC is Native American/American Indian.

The oral health providers administering the MI intervention in this study were DT students from the UMN SOD, as there were no licensed DTs when the study was first initiated. Caregivers were recruited for the study when they presented at the NACC for a scheduled or unscheduled well-child appointment. A staff member from the NACC was appointed site coordinator and was responsible for recruitment, informed consent, enrollment, and randomization of participants. Block randomization was used for assignment to control or test group. The principal and co-principal investigators assessing the outcomes were blinded to participant assignment.

Upon enrollment (visit 1), the site coordinator administered the Caries Risk Test (CRT) test. The site coordinator was responsible for scheduling all future well-child visits with a DT student. Appointments were scheduled following the American Academy of Pediatrics’ recommended intervals for well-child visits: 3 to 5 days old, and at one, two, four, six, nine, and 12 months old [20]. Study participants entered the study at one of these time points and subsequent visits were scheduled at the recommended intervals. The duration of the intervention was 1 year. Prior to the study, DT students were trained and calibrated in MI techniques by a professor in the UMN School of Public Health. Students also attended a presentation on the AIAN culture given by the site coordinator. The MI sessions for test participants took place during the second, third, and fourth visits. During each visit, test group participants engaged in MI sessions with a DT student. To prompt discussion, the DTs used CRT results, Parental Care of Child's Teeth (PCCT) questionnaire results, as well as questions concerning caries risk and protective factors such as use of fluoride and other anti-caries agents, current and past caries activity, snacking habits, etc. Based on the test participant’s answers to the PCCT questionnaire and CRT test results, preventive recommendations were provided to the test participants.

At the second visit, the DT student administered the PCCT questionnaire and conducted the MI session. Three days after the second study visit, the DT student sent a follow-up letter to the test participant affirming strengths to evoke motivation and express confidence in the test participant’s ability to accomplish oral self-care goals and reduce parental risk-related behaviors associated with early childhood caries. DT students made four scheduled telephone calls to the test participant between the second and third visits to monitor progress toward goals, adjust goals, and problem-solve using MI. In the event that a test participant could not be reached by phone, a text message was sent. The third MI session was audiotaped to ensure fidelity of the intervention. The audiotape was evaluated by a MI trainer to determine if there had been any “drift” and provided a written formal report to inform the MI “booster” session. A “booster” MI training session for all DT students was conducted by the person who did the original training. At the fourth visit, the DT student delivered another MI session with the test participant and administered the CRT and PCCT questionnaire. Two weeks after the fourth visit, the DT student telephoned the test participant and provided the results of the CRT test and engaged in discussion using MI strategies. Control participants did not receive oral health education from the DT student, but rather obtained general and oral health information routinely given well-child visits by the primary care physician or nurse based on recommendations formally adopted by the American Academy of Pediatric Dentistry (AAPD). Translators were provided for non-English speaking test and control group participants.

Study instruments used for test and control participants included a CRT and the (PCCT) questionnaire. The CRT was administered at the first and fourth study visit for both test and control group participants [21]. Participants chewed on the chewing gum (wax) provided in the kit for 3 min and spit the mixed saliva into a measuring beaker. The CRT involved salivary samples that were transported under iced conditions. After ultrasonic oscillation, MS levels (MS = Streptococcus Mutans and Streptococcus sobrinus) were assessed through a well-established serial dilution method using bacterial selective media (MSSB) that selected for these bacteria. Lactobacillus were assessed using a selective media of Rogosa Tomato Juice Agar. Colony counting was performed through the use of a gel imaging system (Gel DOc 2000, 50 μm resolution) with a colony counting software program (Quantity One, Biorad). Colony Forming Units (CFU) per ml were calculated from the serial dilution method.

The PCCT questionnaire was developed by Freudenthal and Bowen and was found to be valid and reliable when studying a population of mothers [22]. To facilitate the DT students schedules the PCCT questionnaire was administered at the second and fourth study visit for both test and control participations. The PCCT questionnaire was used in this study to assess demographics and parental oral health behaviors such as child feeding and dietary and oral hygiene practices.

Statistical Analysis System (SAS) version 9.3 was used for data analysis. Descriptive statistics including mean, standard deviation (SD), and frequency were used to summarize the data. A log transformation was utilized to normalize the bacterial load data. The change in mean oral bacterial load was compared between the test and control groups at the fourth visit using a two group t-test. Paired t-tests were used for within group changes. Questions from the Child Feeding and Care Information portion of the PCCT questionnaire was summarized between the two groups for objective two. Two group t-tests (continuous measures) and Fisher’s exact tests (categorical measures) were used to compare instrument questions. *P*-values less than 0.05 were declared statistically significant. For the oral bacterial load outcome (MS and LB), a participant who did not have CRT data at the fourth visit was not included in that analysis.

## Results

Forty-four subjects were initially enrolled in the study. Only the 24 participants who completed all four visits were included in the data analysis (See Flow Diagram). Participant recruitment and all follow-up visits took place during 2011–2013. Table 1 presents test and control participant demographics. In both groups, mothers were reported as the child’s main caregiver (control: 10/11; test: 12/13). Three participants in the control group and one participant in the test group reported both mother and father were the main caregivers. The participant’s average age (SD) was 24.8 (5.4) years for the test group and 30.1 (10.6) years for the control group. Within the test group, 38% of the children were female and 62% were male. Within the control group 64% of the children were female and 36% were male. The average age of the children in the test group was 6 and 7 months for the control group. All participants were predominantly AIAN and from low socioeconomic backgrounds (See Table 1).

**Table 1:** Test and Control Participant Demographics

Racial Categories	n (24) %
American Indian/Alaska Native	20 (83%)
Asian	0 (0%)
Native Hawaiian or other pacific islander	0 (0%)
Black or African American	1 (.04%)
White	0 (0%)
More than one race	3 (7%)
Unknown or not reported	3 (.12%)
Education (highest level completed)	
No formal education	2 (8%)
Some high school	11 (45%)
Completed high school	1 (4%)
Some college	10 (41%)

**Fig. 1:**
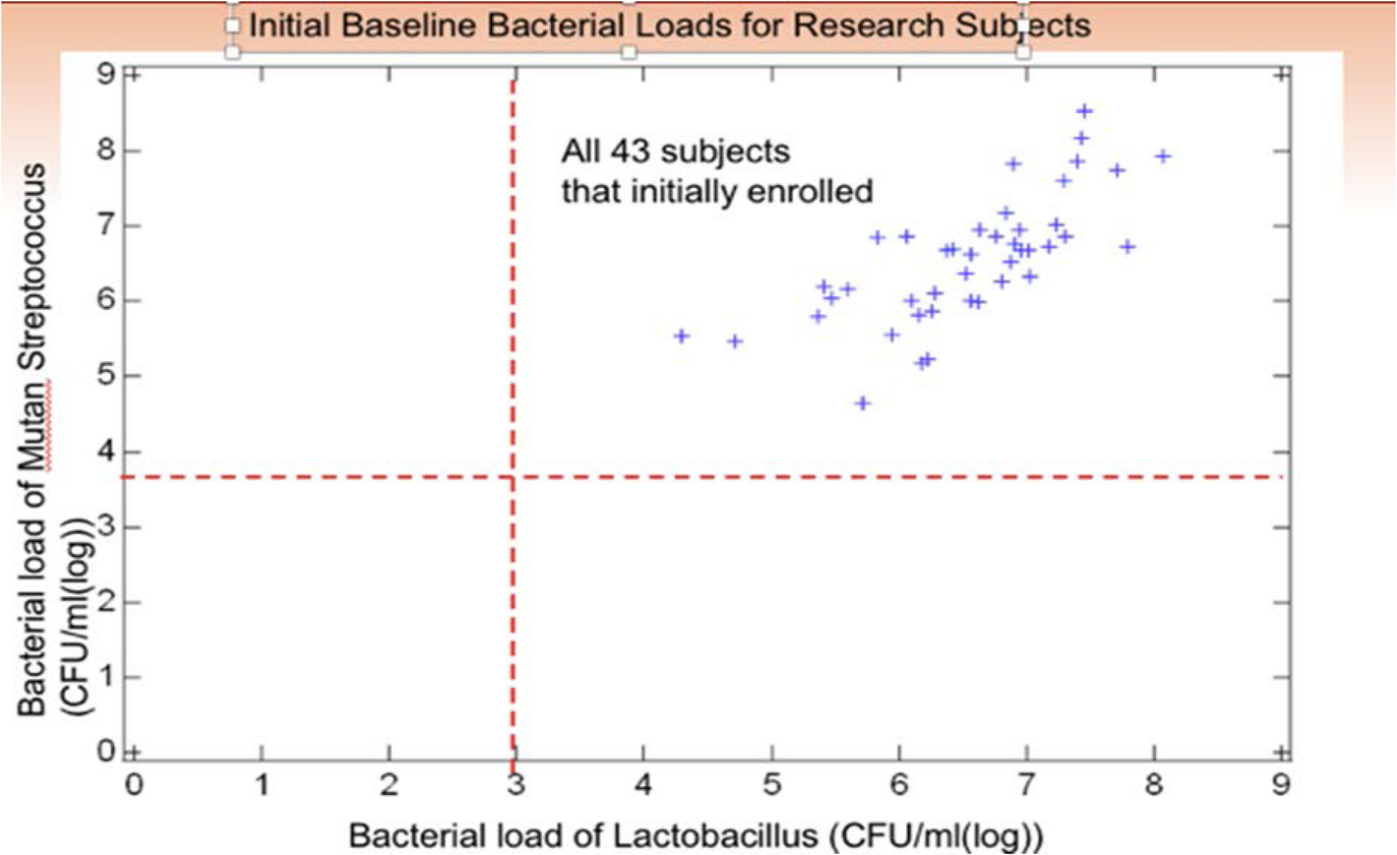
Initial baseline bacterial loads for research subjects

Baseline bacterial levels for LB and MS were similar across groups (See Table 2). At visit one, 15 of the 24 participants self-reported they had toothaches, cavities, or bleeding gums in the past 6 months via the PCCT questionnaire. Results showed initial bacterial levels of MS and LB in the study population were extremely high (Fig. 1). At the last visit, a slight reduction in the mean bacterial levels of LB was observed in both the test and control groups, but not at a level of statistical significance (Table 3). The mean bacterial levels (log CFU/ml) for MS was similar at visit one and four. All paired t-test *p*-values were non-significant (*p* > 0.05). Individual data analysis of risk-related behaviors as measured by the PCCT revealed participants in both the test and control groups slightly decreased ECC risk–related behaviors such as feeding throughout the night and sharing saliva. The test group showed improvement in daily brushing frequency, however, the difference between the test and control groups was not statistically significant (Table 4). The results showed participants possessed oral health knowledge related to feeding practices (See Table 4). Very few participants in either group reported they shared utensils or tasted/chewed the infant’s food during feeding time. The majority of participants did not use sweet snacks to get the child to behave or use sweet snacks as a reward. However, only half of the participants brushed their child’s teeth once or more per day.

**Table 2:** Descriptive Statistics of Bacterial Levels for MS and LB

Group	n	Variable	Mean	SD	Median	Minimum	Maximum
**Treatment**	13	MS (V1)*	6.50	0.72	6.67	5.47	7.75
		MS (V4)	6.52	0.75	6.47	5.36	7.76
		LB (V1)*	6.36	0.86	6.37	4.72	7.71
		LB (V4)	6.25	0.93	6.33	4.10	8.13
**Control**	11	MS (V1)*	6.01	0.75	6.34	4.65	6.86
		MS (V4)	6.46	0.45	6.51	5.47	7.20
		LB (V1)*	6.37	0.80	6.62	4.30	7.03
		LB (V4)	6.04	0.64	6.15	4.40	6.75

*MS* Mutans Streptococci, *LB* Lactobacilli

*V1* visit 1; *V4* visit 4; log CFU/ml

**Table 3:** Change^a^ in Bacterial Levels for MS and LB in CFU/ml^b^

Group	Treatment ***n*** = 13 Mean ± SD	Control ***n*** = 11 Mean ± SD	T-test ***p***-value
**MS**	0.02 ± 0.79	0.44 ± 0.83	0.2131
**LB**	−0.11 ± 0.94	−0.33 ± 0.52	0.4845

^a^Visit 4 minus visit 1

^b^log (base 10) transformed levels of Mutans Streptococci (MS); Lactobacilli (LB)

**Table 4:** Test and Control Participants Responses for ECC Risk Related Behaviors

**Question**		**Visit (V)**	**Treatment** **n = 13** **Mean ± SD**	**Control** **n = 11** **Mean ± SD**	***P*value‡**
How many times a day do you *now* give the child a bottle with something other than water as he/she goes to sleep?		V1	2.7 ± 2.1	2.5 ± 2.7	0.8717
		V4	2.0 ± 1.9	1.8 ± 1.5	0.7966
While awake, how many times a day does the child usually drink or bottle-feed with something other than water as a snack (not including meals)?		V1	2.1 ± 2.2	3.1 ± 2.7	0.3530
		V4	1.4 ± 1.3	2.3 ± 1.7	0.1942
**Question**	**Selected Response**	**Visit (V)**	**Treatment** **n (%)**	**Control** **n (%)**	***P*value‡**
Do you and your child share the same utensils (spoons, forks) during feeding time?	Yes	V1	3 (25%)	3 (27%)	1.0000
		V4	3 (23%)	3 (27%)	1.0000
Do you chew the child’s food or taste it using the child’s utensil before giving it to the child?	Yes	V1	3 (25%)	2 (18%)	1.0000
		V4	2 (15%)	1 (10%)	1.0000
Do you use sweet snacks to get the child to behave?	Never	V1	10 (77%)	8 (89%)	0.6161*/1.0000**
		V4	10 (77%)	7 (78%)	
	Sometimes	V1	3 (23%)	1 (11%)	
		V4	3 (23%)	2(22%)	
Do you use sweet snacks as a reward for the child?	Never	V1	8 (67%)	7 (78%)	0.6594*/1.0000**
		V4	8 (73%)	6 (67%)	
	Sometimes	V1	4 (33%)	2 (22%)	
		V4	3 (27%)	3 (33%)	
How often are the child’s teeth usually cleaned or brushed?	Don’t clean or brush teeth	V1	3 (23)	4 (50%)	0.9096*/0.1435**
		V4	4 (31%)	3 (33%)	
	Less than once a week	V1	0 (0%)	0 (0%	
		V4	0 (0%)	0 (0%)	
	About every other day	V1	1 (8%)	1 (13%)	
		V4	1 (8%)	1 (11%)	
	Almost every day	V1	3 (23%)	1 (13%)	
		V4	0 (0%)	0 (0%)	
	Once a day	V1	2 (15%)	1 (13%)	
		V4	3 (23%)	5 (56%)	
	More than once a day	V1	3 (23%)	1 (13%)	
		V4	5 (38%)	0 (0%)	
	2x week	V1	1 (8%)	0 (0%)	
		V4	0 (0%)	0 (0%)	
Which of the following are used to clean the child’s teeth?	Wash cloth	V1	6 (46%)	3 (33%)	0.6740*/1.0000**
		V4	4 (31%)	3 (30%)	
	Finger brush	V1	2 (15%)	2 (22%)	1.0000*/0.3394**
		V4	4 (31%)	1 (10%)	
	Toothpaste	V1	3 (23%)	1 (11%)	0.6161*/0.4050**
		V4	5 (38%)	2 (20%)	
	Child’s toothbrush	V1	4 (31%)	2 (22%)	1.0000*/1.0000**
		V4	4 (31%)	3 (30%)	
	Shared toothbrush	V1	0 (0%)	0 (0%)	–
		V4	0 (0%)	0 (0%)	
Has the child’s primary caregiver(s) had toothaches, cavities, or bleeding gums in the past 6 months?	Yes	V1	7 (54%)	8 (89%)	0.1649*/1.0000**
		V4	6 (50%)	5 (56%)	

*Visit 1 *p*-value; **Visit 2 *p*-value

‡*P* values are from a two-group t-test for continuous variables and Fisher’s exact tests for the categorical measures. *P* < 0.05 is considered statistically significant. Some subjects did not respond to all questions

## Discussion

This study population had extremely high levels of LB and MS. Even though a reduction in the mean bacterial load for LB was observed in the test group and the control after the last visit, it was not at the level of statistical significance. There was no change in MS levels in either control or test group. The test group showed only minimal improvement in child feeding practices and nighttime bottle habits. Given the success MI has shown in the area of behavior change, we expected different results [17–19]. Yet, research studies conducted after ours show a growing body of evidence that an increase in knowledge does necessarily result in sustained behavior change, especially in high risk populations. Both Freudenthal et al. and Naida et al. showed an effect of improving brushing frequency in mothers who received MI, but did not find any changes in the RAPIDD scores between control and test groups indicating minimal intention to change or sustain new habits [22, 23]. Wilson et al., studied American Indians of the Northern Plains tribe and found both oral health knowledge and behavioral adherence to oral health recommendations to be low [24]. However, behavioral adherence scores were notably lower than knowledge scores. Similarly, participants in our study possessed oral health knowledge; very few participants reported using sweets/snacks as a reward and very few gave their infant sweetened drinks in a bottle. However, knowledge did not translate to improved oral self-care behaviors of the caregivers in the test group. Albino and Tiwari in a review of the literature found only a small number of studies that resulted in behavior change, especially among groups where significant disparities exist [25]. Henshaw and colleagues delivered an MI intervention to primary caregivers in a high-risk population hypothesizing MI would reduce ECC over 2 years as compared with controls [26]. However, Henshaw found MI in combination with other preventive activities resulted in knowledge increases, but did not improve oral health behaviors or affect caries increment [26].

To date, evidence is showing lifestyle decisions and health behaviors are conditioned by cultural and socio-economic context that differs across ethnic and socio-economic groups [14, 24]. The fact that 20 initially enrolled participants did not regularly attend all well-child visits prompts the question that participants’ broader health beliefs and values were not fully understood. Miller and Rollnick assert that for an individual to change, they must feel both confident in their ability to change and believe change is of value to them [16]. Recent research is steering us in the direction of developing interventions focused on bolstering psychosocial strengths that support parents in achieving optimal oral health for themselves and their children [24–27]. Albino et al. assert, “when a child’s oral health is part of a meaningfully organized life, and financial stability is great enough to support their efforts, caregivers are able to ensure their children’s oral health to a greater degree [25].”A greater understanding of social determinants, also known as “upstream variables,” must be considered, as well as underlying beliefs, perceptions and/or attitudes about oral health in the AIAN population, particularly if appropriately targeted preventive interventions are to be developed [26]. Therefore, future investigations should investigate strategies aimed at upstream variables at the community level in addition to targeting individual behavior.

The high level of attrition and resulting small sample size limits the generalizability of our results. Despite gift card incentives, fluoride varnishes and oral self-care products at no cost, a high number of participants did not complete the study. Reasons for the high attrition rate are largely unknown, as several participants disconnected their phone lines so follow-up was not possible. Some participants expressed they did not think they “needed” to attend every well-child visit, as they visited the “healer in their tribe.” Two participants were incarcerated during the study. Using students was also a limitation, as their experience with MI was limited, and their schedules limited their ability to be at NACC full-time and establish relationships within the NACC community. The caries experience of the caregivers in the study was not measured and may have had an effect on the bacterial load. Lastly, social desirability bias may have been a limitation. Given the participants’ high bacterial load and self-reported dental disease, caregivers may have provided biased responses to the PCCT questionnaire reflecting what they believed to be a socially acceptable answer.

## Conclusion

This pilot study investigated the effect of MI on caregivers’ of infants oral self-care behaviors and parental risk-related behaviors for ECC. A slight reduction in the mean bacterial levels of LB was observed in both the test and control groups after the last visit, although not at a level of statistical significance. The mean bacterial load for MS was similar at both visits. MI had minimal effect on oral self-care behaviors as measured by bacterial load, nor did MI reduce parental risk related behavior for ECC in the study population.

## Supplementary information

**Additional file 1.**

**Additional file 2.**

## Data Availability

The datasets used and/or analyzed during the current study are available upon reasonable request from the corresponding author.

## Abbreviations

AAPD: The American Academy of Pediatric Dentistry
AIAN: American Indian and Alaska Native
CAMBRA: Caries Management by Risk Assessment
CRT: Caries risk test
DT: Dental therapy
ECC: Early childhood caries
FQHC: Federally Qualified Health Center
IRB: Institutional Review Board
LB: Lactobacilli
MI: Motivational interviewing
MS: Mutans streptococci
NAAC: Native American Community Clinic
PCCT: Parental Care of Child’s Teeth
RAPIDD: Readiness Assessment of Parents concerning Infant Dental Decay
SAS: Statistical Analysis System
SD: Standard deviation
SES: Socioeconomic status
SOD: School of Dentistry
UMN: University of Minnesota
US: United States

## References

[1] Çolak H, Dülgergil ÇT, Dalli M, Hamidi MM (2013). Early childhood caries update: a review of causes, diagnoses, and treatments. J Nat Sci Biol Med.

[2] Young DA, Featherstone JD, Roth JR (2007). Caries management by risk assessment: implementation guidelines. J Calif Dent Assoc.

[3] Isong IA, Luff D, Perrin JM (2012). Parental perspectives of early childhood caries. Clin Pediatr.

[4] Relvas M, Coelho C, Velazco Henriques C (2014). Cariogenic bacteria and dental health status in adolescents: the role of oral health behaviours. Eur J Paediatr Dent.

[5] Oral Health Care During Pregnancy Expert Workgroup (2012). Oral health care during pregnancy: a National Consensus Statement—Summary of an expert workgroup meeting.

[6] Esin S, Pasini M, Miceli M (2018). Longitudinal study on the effect of oral hygiene measures on the salivary count of microbial species with cariogenic potential. J Biol Regul Homeost Agents.

[7] Azevedo M, Romano A, Correa M, Santos IS, Cenci M (2015). Evaluation of a feasible educational intervention in preventing early childhood caries. Braz Oral Res.

[8] Bugis BA. Early childhood caries and the impact of current U.S. Medicaid program: an overview. Int J Dent. 2012;2012:348237. 10.1155/2012/348237 PMC3312229 22496690

[9] Office of Revisor of Statutes, Minnesota. Minnesota Board of Dentistry Statutes; 2010 [Chapters 150A and 319B].

[10] Phipps KR, Ricks RL: The oral health of American Indian and Alaska Native children aged 1–5 years: results of the 2014 IHS Oral Health Survey. Indian Health Service data brief; 2015. [https://www.ihs.gov/doh/documents/IHS_Data_Brief_1-5_Year-Old.pdf] Accessed 6 June 2017.

[11] Schroth RJ, Halchuk S, Star L (2013). Prevalence and risk factors of caregiver reported severe early childhood caries in Manitoba first nations children: results from the RHS phase 2 (2008–2010). Int JCircumpolar Health.

[12] Robertson LD, Phipps KR, Oh J, Loesche WJ, Kaciroti N, Symington JM (2012). Using chlorhexidine varnish to prevent early childhood caries in American Indian children. J Public Health Dent.

[13] Ismail AI, Ondersma S, Jedele JMRJ, Little RJ, Lepkowski JM (2011). Evaluation of a brief tailored motivational intervention to prevent early childhood caries. Community Dent Oral Epidemiol.

[14] Harrison R, Veronneau J, Leroux B. Effectiveness of maternal counseling in reducing caries in cree children. J Dent Res. 2012:1032–7. 10.1177/0022034512459758 22983408

[15] Colvara BC, Faustino-Silva DD, Meyer E, Hugo FN, Hilgert JB, Celeste RK (2018). Motivational interviewing in preventing early childhood caries in primary healthcare: a community-based randomized cluster trial. J Pediatrics.

[16] Miller WR, Rollnick S (2013). Motivational interviewing helping people change.

[17] Lindson-Hawley N, Thompson TP, Begh R (2015). Motivational interviewing for smoking cessation. Cochrane Database Syst Rev.

[18] Resnicow K, McMaster F, Bocian A, Harris D, Zhou Y, Snetselaar L (2015). Motivational interviewing and dietary counseling for obesity in primary care: an RCT. Pediatrics..

[19] Neff JA, Walters ST, Braitman AL, Kelley ML, Paulson JF, Brickhouse TH (2013). A brief motivational intervention for heavy alcohol use in dental practice settings: rationale and development. J Health Psychol.

[20] American Academy of Pediatric Dentistry (2015). Policy on the dental home. Pediatr Dent.

[21] Cannon M, Trent B, Vorachek A (2013). Effectiveness of CRT at measuring the salivary level of bacteria in caries prone children with probiotic therapy. J Clin Pediatr Dent.

[22] Freudentahl J, Bowen BM (2010). Motivational interviewing to decrease parental risk-related behaviors for early childhood caries. J Den Hyg.

[23] Naidu R, Nunn J, Irwin JD (2015). The effect of motivational interviewing on oral healthcare knowledge, attitudes, and behaviour of parents and caregivers of pre-school children: an exploratory cluster randomised controlled study. BMC Oral Health.

[24] Wilson A, Brega A, Batliner TS, Henderson W, Campagna MS, Fehringer K, Gallegos J, Daniels D, Albino J (2014). Assessment of prenatal Oral health knowledge and behaviors among American Indians of a North Plains tribe. J Public Health Dent.

[25] Albino J, Tiwari T (2016). Preventing childhood caries a review of recent behavioral research. J Dent Res.

[26] Henshaw MM, Borrelli B, Gregorich SE, Heaton B, Tooley EM, Santo W, Cheng NF, Rasmussen M, Helman S, Shain S, Garcia RI (2018). Randomized trial of motivational interviewing to prevent early childhood caries in public housing. JDR Clin Trans Res.

[27] Albino J, Tiwari T, Henderson WG, Thomas JF, Braun PA, Batliner TS (2018). Parental psychosocial factors and childhood caries prevention: data from an American Indian population. Community Dent Oral Epidemiol.

