# Supplementary figures and images for: Using motivational interviewing to reduce parental risk related behaviors for early childhood caries: a pilot study
Source: BMC Oral Health. 2020 Mar 29;20:90. doi: 10.1186/s12903-020-1052-6 (PMC7104483; doi:10.1186/s12903-020-1052-6)

## Flow Diagram

### Reducing Parental Risk Related Behaviors for ECC

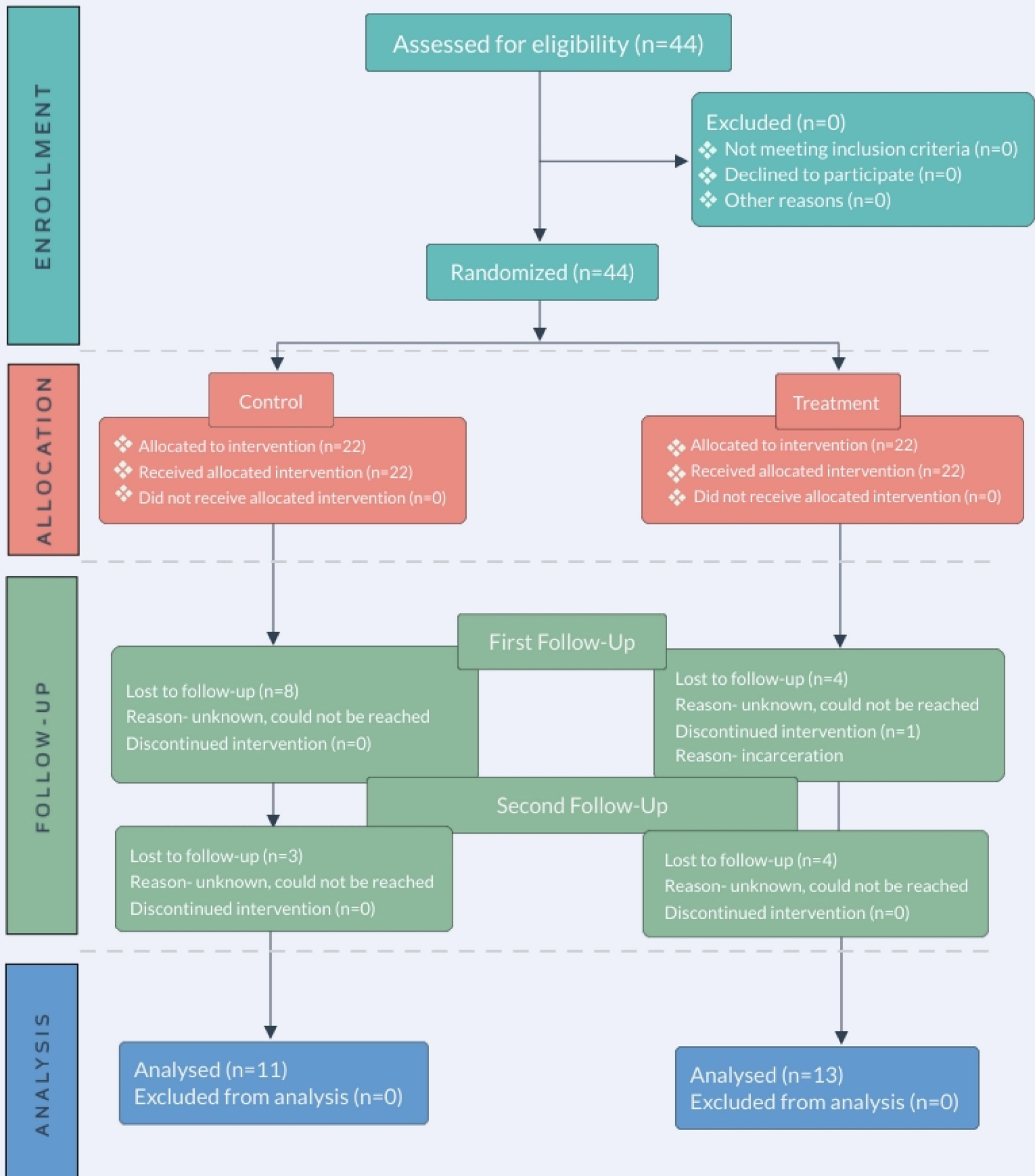

Supplement: Supplementary file 1 — Additional file 1. [file 12903_2020_1052_MOESM1_ESM.pdf]
