# Supplementary material for: Using motivational interviewing to reduce parental risk related behaviors for early childhood caries: a pilot study
Source: BMC Oral Health. 2020 Mar 29;20:90. doi: 10.1186/s12903-020-1052-6 (PMC7104483; doi:10.1186/s12903-020-1052-6)
Supplement: Supplementary file 2 — Additional file 2. [file 12903_2020_1052_MOESM2_ESM.doc]

# **Appendix A: Parental Care of Child’s Teeth Questionnaire**

# **Directions: Please answer the following questions or circle the response(s) that apply.**

# Demographic Information

# Child’s date of birth __________________________________

# Month Year

# Child’s sex? (circle one) boy girl

# Who is the child’s main caregiver? (circle all that apply)

# child’s mother child’s father

# child’s grandparent child’s aunt

# child’s uncle child’s sibling(s)

# other (please specify)____________________________

# 4. Who helps you take care of the child? (circle all that apply)

# no one else

# your husband or partner

# your mother

# your grandparent

# your relative or friend

# your child’s sibling(s)

# day care (babysitter)

# other (please specify)

# How many other children live in the child’s household? ____________________

# What are their ages?___________________________________________

# Child’s mother’s date of birth _________________________________________

# Month Year

# What is the highest level of education that the mother of the child has completed?

# (circle one)

# No formal education

# Some high school

# Completed high school

# Some college

# Completed bachelors degree or higher

# Child Feeding and Care Information

# What types of feeding or food consumption occurs in your child’s normal day?

# (circle all that apply)

# breast feeding solid food

# bottle feeding cup for drinking

# soft food only

# How many times a day do you **now** give the child a bottle with something other than

# water as he/she goes to sleep? (circle one)

# 0 1 2 3 4 5 6 7 8 9 10 or more

# 10. While **awake**, how many times a day does the child usually drink or bottle-feed with something other than water as a snack (not including meals)? (circle one)

# 0 1 2 3 4 5 6 7 8 9 10 or more

# 11. Do you and your child share the same utensils (spoons, forks) during feeding

# time?

# Yes No

# 12. Do you chew the child’s food or taste it using the child’s utensil before giving it

# to the child?

# Yes No

# How many times a day does the child usually: (circle one)

# Eat a morning snack?

# 0 1 2 3 4 5

#

# Eat an afternoon snack?

# 0 1 2 3 4 5

#

# Eat or drink a snack after dinner or before bedtime?

#

# 0 1 2 3 4 5

# Circle the most common **foods** that the child eats as snacks?

# Banana Pasta, potatoes

# Cereal Cookies, donuts

# Crackers Chips

# Applesauce Yogurt, Jello

# Vegetables Fresh fruit (not banana)

# Cheese Peanut butter, nuts

# Candy Raisins, jam

# Other ___________________________________________________________

# Circle the most common **drinks** the child has as snacks?

# Milk (plain) Water

# Milk (sweetened) Sweetened drinks

# Kool aid Fruit juice

# Soda pop Lemon aid

# Other ____________________________________________________________

# Do you use sweet snacks to get the child to behave?

# Never Sometimes Usually Almost Always

# 17. Do you use sweet snacks as a reward for the child?

# Never Sometimes Usually Almost Always

# 18. How often are the child’s teeth usually cleaned or brushed? (circle one)

# Don’t clean or brush teeth

# Less than once a week

# About every other day

# Almost every day

# Once a day

# More than once a day

# Which of the following are used to clean the child’s teeth? (circle all that apply)

# wash cloth child’s own toothbrush

# finger brush a shared toothbrush

# toothpaste other (please specify)________________________

# 20. Has the child’s primary caregiver(s) had toothaches, cavities, or bleeding gums in

# the past six months?

# Yes No
